# Symptomatic Trigeminal Autonomic Cephalalgias

**DOI:** 10.1007/s11916-015-0514-z

**Published:** 2015-06-20

**Authors:** Ilse F. de Coo, Leopoldine A. Wilbrink, Joost Haan

**Affiliations:** Department of Neurology Leiden University Medical Centre, Leiden, The Netherlands; Department of Neurology Alrijne Hospital, Leiderdorp, The Netherlands

**Keywords:** Cluster headache, Trigeminal autonomic cephalalgia, Paroxysmal hemicrania, Hemicrania continua, Short-lasting unilateral neuralgiform headache with conjunctival tearing, Short-lasting unilateral neuralgiform headache attacks with cranial autonomic symptoms

## Abstract

Trigeminal autonomic cephalalgias (TACs) are primary headache syndromes that share some clinical features such as a trigeminal distribution of the pain and accompanying ipsilateral autonomic symptoms. By definition, no underlying structural lesion for the phenotype is found. There are, however, many descriptions in the literature of patients with structural lesions causing symptoms that are indistinguishable from those of idiopathic TACs. In this article, we review the recent insights in symptomatic TACs by comparing and categorizing newly published cases. We confirm that symptomatic TACs can have typical phenotypes. It is of crucial importance to identify symptomatic TACs, as the underlying cause will influence treatment and outcome. Our update focuses on when a structural lesion should be sought.

## Introduction

Trigeminal autonomic cephalalgias (TACs) are primary headache syndromes that owe their name to the trigeminal distribution of the pain and the accompanying ipsilateral autonomic symptoms, as defined by the International Classification of Headache Disorders (ICHD)-III beta criteria [1]. The most prevalent TAC is cluster headache, but the category also includes rare diseases such as paroxysmal hemicrania, short-lasting unilateral neuralgiform headache attacks with conjunctival injection and tearing (SUNCT), short-lasting unilateral neuralgiform headache attacks with cranial autonomic symptoms (SUNA), and hemicrania continua.

It is well known that an underlying structural lesion can lead to TAC symptoms, which cannot easily be differentiated from those of idiopathic TACs [2, 3•]. Recognizing these underlying pathologies is of crucial importance, as they can influence treatment and outcome. Here, we will give an update of recently published cases with an underlying structural lesion and a TAC phenotype.

## Methods

In 2009, our group published a comprehensive update of symptomatic TACs, reported until mid-February 2009 [4]. With this report as starting point, we conducted a PubMed search from February 2009 to January 2015 with the following key words: trigeminal autonomic cephalalgia, cluster headache, hemicrania continua, SUNCT, SUNA, paroxysmal hemicrania, secondary, and symptomatic. Only articles written in English were included of which the full text was available. Cases were divided into three categories: *probably secondary*, *possibly secondary*, and *unknown*. Cases were defined as *probably secondary* when there was a dramatic improvement of the headache after treatment of the underlying lesion. Cases were defined as *possibly secondary* when the patient was treated but did not become headache free, or was not treated, but where a causal relation was possible based on previous experience with other patients. Efficacy of indomethacin was not considered as treatment response in paroxysmal hemicrania and hemicrania continua, as this is one of the diagnostic criteria and not specifically aiming at an underlying lesion. The category *unknown* was used for patients in which a causal relation between the phenotype and the lesion was less likely or at least unclear: in most cases, the patient was not treated and a causal relation between the lesion and the TAC was unlikely on anatomical grounds and/or a probable incidental finding.

## Results

### Cluster Headache

We found 23 cases with a cluster headache-like phenotype in 23 articles [2, 5, 6•, 7–10, 11•, 12–26]. We excluded 3 patients, as they did not fulfil the ICHD-III criteria beta version, all having an attack duration of more than 3 h [18, 23, 25]. We excluded also another patient who did not have a structural lesion [16]. This resulted in 19 patients of whom 12 could be categorized as *probably secondary* and 7 as *possibly secondary* (Table 1).

**Table 1:** Symptomatic cases of cluster headache

Authors	Publication year	Age (year)	Sex	Headache phenotype	Duration of CH symptoms	Atypical features	Underlying lesion	Treatment	Follow-up	Outcome
Probably symptomatic cluster headache										
Edvardsson [13]	2014	49	M	Cluster headache	1 month	Nausea, photophobia, and phonophobia	Non-functioning pituitary adenoma (chromophobe adenoma)	Sumatriptan SC, oxygen, verapamil, surgery	17 months	Pain free
Malissart et al. [19]	2014	60	F	Cluster headache	3 days	–	Ipsilateral carotid paraganglioma	Surgery	Unknown	Pain free
Edvardsson et al. [10]	2013	43	M	Cluster headache	2 months	Nausea and photophobia/phonophobia	Intrasellar arachnoid cyst	Craniotomy with cyst fenestration	4 months	Pain free
Edvardsson et al. [12]	2013	21	M	Cluster headache	3 weeks	–	Maxillary sinusitis	Antibiotics and sinus puncture	4 years	Pain free
Levy et al. [17]	2012	25	M	Cluster headache	3 months	–	Ipsilateral pituitary macroprolactinoma	Cabergoline	Unknown	Pain free
Edvardssonet al. [9]	2012	41	M	Cluster headache	3 months	Nausea and photophobia/phonophobia	Ipsilateral glioblastoma multiforme	Surgery	12 months	Pain free
Ranieri et al. [22]	2009	39	M	Cluster headache	14 years	Maxillary pain next to periorbital pain, continues daily pain during last 7 months, tooth grinding, and frequently waking up at night	Obstructive sleep apnoea diagnosis 14 years after CH diagnosis	Intra-oral device	12 months	Pain free
Sewell et al. [24]	2009	34	M	Cluster headache	17 years	At moment of consultation, restless legs syndrome and numbness in fingers	Stroke caused by moyamoya	Two cranial bypasses	6 years	Pain free
Edvardsson [11•]	2013	24	M	Cluster headache	4 weeks	–	Acute maxillary sinusitis	Antibiotics and sinus puncture	Several years	Pain free
Fontaine et al. [14]	2013	27	M	Cluster headache	4 months	–	Ipsilateral hemangiopericytoma	Surgery	9 months	Pain free
Van der Vlist et al. [26]	2013	31	M	Cluster headache	2 months	Diffuse headache next to the attacks	Sarcoidosis (also hypothalamic lesion)	Prednisone course	7 months	Pain free
Créac’h et al. [8]	2010	44	F	Cluster headache	7 months	Trigger factor: rotation of head to the right	Neurovascular compression caused by fibrosis surrounding both C3 and right vertebral artery	Verapamil for 6 months, microvascular dissection	2.5 years	Pain free
Possibly symptomatic cluster headache										
Candeloro et al. [6•]	2013	39	M	Cluster headache	21 years	Once attack duration of >3 h	Dissection of the right distal internal carotid artery	Heparin	6 months	Unknown
Mijajlović et al. [21]	2014	45	M	Cluster headache	7 days	–	Multiple sclerosis	Methylprednisolone course with afterwards verapamil for 1 year	3 years	Pain free
Gil-Gouveia et al. [15]	2013	79	F	Cluster headache	–	–	48 h after lens phacoemulsification and intraocular lens implant	Verapamil, sodium valproate, oxygen	9 months	Decrease in attack frequency
Messina et al. [20]	2013	27	M	Cluster headache	–	–	Angiomyolipoma	Hypothalamic deep brain stimulation	Unknown	Decrease in attack frequency
Donat [2]	2011	33	M	Cluster headache	–	–	Multiple sclerosis	Verapamil	10 months	Pain free
Choi et al. [7]	2009	52	F	Cluster headache	10 years	Attacks sometimes on both sides. This time also blurred vision and central horizontal scotoma	Recurrent posterior scleritis and aseptic meningitis	Prednisone course	2 months	Unknown
Benitez-Rosario et al. [5]	2009	41	M	Cluster headache	12 months	Depressive symptoms	Ipsilateral macroprolactinoma	Cabergoline, hormonal replacement, prednisone course, verapamil	About 1–2 months	Pain free

Of the 12 cases in the *probably secondary* category, 5 had a neoplasm [15, 19, 21, 27, 28]: a non-functioning pituitary adenoma, an ipsilateral carotid paraganglioma, an ipsilateral prolactinoma, an ipsilateral glioblastoma multiforme, and an ipsilateral hemangiopericytoma. A vascular cause, a stroke secondary to moyamoya disease, was found in 1 patient [29]. Other patients had an intrasellar arachnoid cyst, maxillary sinusitis (*n* = 2), compression of the right vertebral artery by fibrosis, sarcoidosis (with a hypothalamic lesion), and obstructive sleep apnoea [8, 10, 11•, 12, 22, 26].

There were seven cases defined as *possibly secondary*. Multiple sclerosis was found in two, of whom both became pain free under verapamil or prednisone, which are used as prophylactic cluster headache medication and therefore are not strictly aiming at the *underlying lesion* [2, 21]. Another patient had an internal carotid artery dissection, but the outcome after treatment remained unclear [6•]. Other diagnoses in this category are as follows: recurrent posterior scleritis and a specific meningitis (treated with prednisone), post-operative cluster headache (lens phacoemulsification and intraocular lens implant), an angiomyolipoma, and an ipsilateral macroprolactinoma [5, 7, 15, 20]. The latter two patients responded completely or partly to treatment of the underlying lesion, but only in combination with preventive cluster headache treatment.

### Paroxysmal Hemicrania

We identified three cases of paroxysmal hemicrania, of whom all were excluded as they did not fulfil the ICHD-III criteria beta version [27, 30, 31]. The missing criterion in two patients was an unknown response to indomethacin [30, 31], and the third reported bilateral instead of unilateral facial pain [27].

### Hemicrania Continua

We identified seven cases [28, 32–35] of symptomatic hemicrania continua of whom one was excluded as the patient did not receive indomethacin [28]. We categorized two cases as *probably symptomatic*, three as *possibly symptomatic*, and one as *unknown* (Table 2).

**Table 2:** Symptomatic cases of hemicrania continua

Authors	Publication (year)	Age (year)	Sex	Headache phenotype	Duration symptoms	Indomethacin response	Atypical features	Underlying lesion	Treatment	Follow-up	Outcome
Probably symptomatic hemicrania continua											
Mathew et al.[33]	2014	42	M	Hemicrania continua	1 month	Completely	Duration <3 months	Cerebral venous thrombosis	Anticoagulation and antiedema therapy	3 days	Pain free
Robbins et al.[35]	2010	55	F	Hemicrania continua	7 months	Completely	Ocular foreign body sensation	Brain metastases of primary lung adenocarcinoma	Indomethacin for 3 days, dexamethasone, chemotherapy, and whole brain radiation	6 months	Pain free
Possibly symptomatic hemicrania continua											
Prakash et al.[34]	2009	52	M	Hemicrania continua	10 years	Completely	–	Post-traumatic	Indomethacin	15 months	Pain free
Prakash et al.[34]	2009	36	F	Hemicrania continua	2 years	Completely	–	Post-operative (tubectomy)	Indomethacin	5 months	Pain free
Prakash et al.[34]	2009	44	F	Hemicrania continua	1 year	Completely	–	Post-operative (left parietal craniotomy for evacuation of haematoma and repair of the fracture after trauma)	Indomethacin	10 months	Pain free
Unknown: symptomatic hemicrania continua or incidental co-finding											
DeLange et al.[32]	2014	55	F	Hemicrania continua	4 months	Completely	Disc edema, visual symptoms	Orbital pseudotumour	Prednisone, indomethacin	Unknown	Pain free

The underlying lesions in the cases defined as *probably symptomatic* were a cerebral venous thrombosis and brain metastases of a primary lung adenocarcinoma [33, 35]. Both patients responded to treatment of the underlying cause, and indomethacin could be withdrawn.

In patients defined as *possibly symptomatic*, the possible causes were post-traumatic and twice post-operative [34]. All received indomethacin as treatment for their hemicrania continua.

One case was classified as *unknown*. This patient was diagnosed with an orbital pseudotumour, treated with prednisone and indomethacin [32].

### SUNCT and SUNA

We found 29 cases of SUNCT and SUNA [3•, 29, 36–48] of whom 1 was excluded because of bilateral pain during the attacks [48]. There were 14 cases defined as *probably symptomatic*, 12 as *possibly symptomatic*, and 2 as *unknown* (Table 3).

**Table 3:** Symptomatic cases of SUNCT/SUNA

Authors	Publication (year)	Age (year)	Sex	Headache phenotype	Duration symptoms	Atypical features	Underlying lesion	Treatment	Follow-up	Outcome
Probably symptomatic SUNCT/SUNA										
Favoni et al.[49]	2013	53	F	SUNCT	3 years	–	Compression of trigeminal nerve by right superior cerebellar artery	Microvascular decompression trigeminal nerve	11 months	Pain free
Chitsanikul et al. [37]	2013	45	M	SUNCT	3 years	Improvement by vigorous activity	Ipsilateral mixed gangliocytoma and pituitary adenoma	Surgery	4 years	Pain free
Chitsanikul et al. [37]	2013	51	F	SUNCT	4 years	Right arm and facial numbness during attacks, irregular menstruation, decrease in libido, galactorrhoea	Ipsilateral prolactinoma	Surgery	18 months	Improvement in frequency and intensity
Cöven et al. [38]	2013	57	F	SUNCT	3 years	–	Aneurysm	Surgery	Unknown	Pain free
Domingos et al. [40]	2012	46	M	SUNCT	3 months	Blurred vision outside attack	Cavernous sinus dural fistula	Surgery	1 year	Pain free
Guerreiro et al. [42]	2009	57	M	SUNCT	3 months		Compression trigeminal nerve by superior cerebellar artery	Microvascular decompression	Unknown	Pain free
De Lourdes et al. [39]	2009	50	M	SUNCT	4 years	–	Ipsilateral macroprolactinoma	Cabergoline	7 months	Pain free
Rodgers et al. [46]	2013	33	M	SUNCT	6–8 months	Triggered by head movements, chewing, jaw opening	Ipsilateral epidermoid tumour in cerebellopontine angle	Gabapentin, duloxetine, pregabalin, phenobarbital, morphine, steroids, carbamazepine all ineffective, afterwards surgery	6 months	Pain free
Williams et al. [3•]	2010	71	M	SUNCT	6 years		Compression trigeminal nerve by superior cerebellar artery	Lamotrigine, carbamazepine, gabapentin, baclofen, and prednisolone without benefit, surgery	32 months	Pain free
Williams et al. [3•]	2010	54	M	SUNCT/SUNA	1–2 months		Compression trigeminal nerve by superior cerebellar artery	Carbamazepine, phenytoin, gabapentin, and baclofen without benefit, surgery	32 months	Pain free
Williams et al. [3•]	2010	46	M	SUNCT	3 years		Compression trigeminal nerve by anterior inferior cerebellar artery, vein, adhesions	Lamotrigine, valproic acid, and topiramate without benefit, surgery	30 months	Pain free
Williams et al. [3•]	2010	56	M	SUNA	1 year		Compression trigeminal nerve by superior cerebellar artery	Lamotrigine and carbamazepine without benefit, surgery	20 months	Pain free
Williams et al. [3•]	2010	51	F	SUNCT	5 years		Compression trigeminal nerve by superior cerebellar artery	Lamotrigine, prednisolone, and morphine without benefit, surgery	10 months	Pain free
Williams et al. [3•]	2010	49	M	SUNCT	26 years	–	Compression trigeminal nerve by superior cerebellar artery	Lamotrigine and topiramate without benefit, surgery	9 months	Pain free
Possibly symptomatic SUNCT/ SUNA										
Favoni et al. [49]	2013	55	M	SUNCT	9 years	–	Compression trigeminal nerve by superior cerebellar artery	Gabapentin, verapamil, pregabapentin, and iv corticosteroids course, indomethacin for 1 month without effect, response on carbamazepine	Unknown	Pain free
Chitsanikul et al. [37]	2013	25	F	SUNCT	6 years	–	Ipsilateral prolactinoma	Indomethacin, lamotrigine, topiramate, carbamazepine, gabapentin, oxycodone, and greater occipital nerve block all without effect, surgery	1 year	No improvement
Chitsanikul et al. [37]	2013	56	F	SUNCT	–	–	Ipsilateral pituitary tumour	Surgery	6 months	No improvement
Chitsanikul et al. [37]	2013	30	F	SUNCT	12 years	–	Ipsilateral prolactinoma	Surgery	20 years	No improvement
Cascella et al.[29]	2011	57	F	SUNCT	1 month	–	Lung adenocarcinoma	Greater occipital nerve block and indomethacin without effect, valacyclovir, and prednisone course, chemotherapy, gabapentin	5 months	Pain free
Kutschenko et al. [44]	2010	81	F	SUNCT	5 months	–	Ipsilateral meningioma	Gabapentin	Unknown	Pain free
Bogorad et al. [36]	2010	61	F	SUNCT	2 years	–	Multiple sclerosis	Carbamazepine, steroids, and indomethacin	1 day	Pain free
Theeler et al. [47]	2009	27	F	SUNCT	14 years	Abnormal menstrual cycles and galactorrhea	History of left optical nerve hypoplasia since 2 years, mild hypothalamic-pituitary dysfunction	Observation	8 months	No change in attack frequency
Ito et al. [43]	2009	49	M	SUNCT	Several days	Fever	Viral meningitis	Sumatriptan SC for 3 days	Unknown, at least 4 days	Pain free
Williams et al. [3•]	2010	61	M	SUNCT/SUNA	3 years		Compression trigeminal nerve by superior cerebellar artery and vein	Lamotrigine and phenytoin without benefit, surgery	22 months	Persistent attacks
Williams et al. [3•]	2010	48	F	SUNA	2 years		Compression trigeminal nerve by anterior inferior cerebellar artery and vein	Lamotrigine and gabapentin without benefit, surgery	20 months	Persistent attacks
Williams et al. [3•]	2010	49	F	SUNCT	5 years		Compression trigeminal nerve by superior cerebellar artery	Lamotrigine, indomethacin, pethidine, and topiramate without benefit, surgery	10 months	Persistent attacks
Unknown: symptomatic SUNCT/SUNA or incidental co-finding										
Granato et al. [41]	2014	72	M	SUNCT	–	Fever	Varicella zoster virus meningoencephalitis (after 1 week)	Gabapentin, acyclovir intravenous course, anti-platelet treatment	1 month	Died
Panconesi et al. [45]	2009	54	M	SUNCT or trigeminal neuralgia	14 years	–	Posterior fossa abnormality	Gabapentin together with carbamazepine	Unknown	Reduction in attacks

*SUNCT* short-lasting unilateral neuralgiform headache attacks with conjunctival injection and tearing, *SUNA* short-lasting unilateral neuralgiform headache attacks with cranial autonomic symptoms

Most cases were defined as *probably symptomatic* SUNCT/SUNA. The cause found in patients with *probably symptomatic* SUNCT was most often compression of the trigeminal nerve by an artery (8 out of 14), followed by malignancies as a mixed gangliocytoma, an epidermoid tumour, and prolactinomas [3•, 37, 39, 42, 46, 49]. Furthermore, an aneurysm and cavernous sinus dural fistula were found [38, 40]. All patients responded completely to treatment of the underlying cause, which was most often surgery.

Tumours were most often the underlying cause in the category *possibly symptomatic* SUNCT/SUNA: an ipsilateral prolactinoma (*n* = 2), an ipsilateral pituitary tumour, a lung adenocarcinoma, and an ipsilateral meningioma [29, 37, 44]. Furthermore, trigeminal nerve compression (*n* = 4), multiple sclerosis, a mild hypothalamic-pituitary dysfunction by optical nerve hypoplasia, and a viral meningitis were reported. Five patients became pain free under preventive SUNCT treatment [3•, 36, 43, 47, 49].

There were two cases categorized as *unknown*. One patient developed a varicella zoster virus meningoencephalitis 1 week after the SUNCT attacks and died within several weeks from arrhythmia secondary to myocarditis, likely as consequence of the viremia [41]. In the other patient, a small posterior skull and a cerebellar hypoplasia, without dysplasia, were found. A causal relation between the development of SUNCT and this anomaly is uncertain [45].

## Conclusion

The goal of this review was to give an update on underlying structural lesions associated with TACs, published between February 2009 (since the last review) and January 2015. We identified 53 typical cases: 19 cases with cluster headache, no cases with paroxysmal hemicrania, 6 cases with hemicrania continua, and 28 cases with SUNCT/SUNA.

Tumours were reported in 16 of the 53 cases diagnosed with a TAC, mainly pituitary tumours. Prolactinomas were found in 2 cluster headache and 4 SUNCT patients, followed by pituitary adenomas (*n* = 2). It has indeed been reported that pituitary tumours account for a large portion of the secondary causes of SUNCT [50]. The other way around, various types of headache including TACs have been reported as a frequent symptom of pituitary tumours [51]. An association between the side of the tumour and side of the headache has been suggested [52••]. In most of the reported cases of secondary SUNCT and secondary cluster headache, surgery or medical treatment of the pituitary tumour resulted in improvement.

A vascular lesion as an underlying cause was less often found. An intracranial or extracranial dissection was reported in only 1 of the 19 cluster headache patients. This patient was diagnosed with cluster headache several years before he experienced a cluster headache attack with prolonged duration, which was probably caused by a carotid dissection [6•]. Dissection as a cause for cluster headache is rare but has been reported in earlier reviews [4, 50]. Recognition is of crucial importance as it can have serious consequences for patients. Cases with carotid dissection have shown improvement of the headache after antiaggregant or anticoagulant therapy. Most patients did not even need preventive cluster headache treatment. Repeated contrast-enhanced magnetic resonance imaging (MRI) should be considered if the characteristics of the headache attacks change over time.

In 12 SUNCT patients, a trigeminal nerve compression by vascular structures as possible cause of SUNCT was found. Eight of 11 surgically treated patients became headache free, whereas only 3 patients had no benefit of the procedure. This is an important finding as SUNCT is often considered medically intractable. Trigeminal nerve compression was found in 42.8 % of this series.

A sinusitis was considered probably causal in 2 cluster headache patients. Sinusitis is a common misdiagnosis in cluster headache. Lainez et al. showed that 14 of 75 cluster headache patients (18.7 %) were initially misdiagnosed as having a sinusitis [53••]. It is sometimes very difficult to make a clear distinction between sinusitis and a TAC [54].

In summary, we found 53 typical cases of secondary TACs in our literature study covering the period from February 2009 to January 2015. Secondary underlying lesions seem to be rare in TACs. However, physicians should be aware of possible underlying pathology, as, for example, prolactinomas or glioblastomas, arteriovenous malformations, dissections, and various inflammations can cause a TAC-like phenotype. In our opinion, not only a contrast-enhanced cerebral MRI should be considered once in every patient to exclude a causal underlying pathology but also imaging of cervical vascular structures.

Most of our findings are in accordance with those of Wilbrink et al. [4]. Of additional importance is the more recent observation that in more than 40 % of patients with SUNCT/SUNA, a trigeminal nerve compression by the superior or inferior cerebellar artery was present and that most of these patients experienced spectacular improvement of their headache after surgical decompression. In contrast to other reviews, we found less frequently an intracranial or extracranial dissection causing cluster headache [4, 50]. This could be explained by the fact that there are already various case reports about intracranial and extracranial dissections causing cluster headache [55–57]. The importance of a cerebral MRI to exclude underlying lesions is shown in the current review, as cerebral lesions (e.g. pituitary tumours) were associated with TACs.

## Abbreviations

TACs: Trigeminal autonomic cephalalgias
ICHD: International Classification of Headache Disorders
SUNCT: Short-lasting unilateral neuralgiform headache with conjunctival tearing
SUNA: Short-lasting unilateral neuralgiform headache attacks with cranial autonomic symptoms
MRI: Magnetic resonance imaging
